# Familial Multiple Trichoepitheliomas Across Three Generations With a Pathogenic CYLD Variant Identified in One Affected Family Member: A Case Report

**DOI:** 10.7759/cureus.117938

**Published:** 2026-10-14

**Authors:** Carla Genevcius, Annelise C Simines, Giovana B Cocco, Clara C Dorigheli, Maysa M Lima, Mayara M Taura

**Affiliations:** 1 Dermatology, Fundação Educacional do Município de Assis, Assis, BRA

**Keywords:** adnexal tumors, cyld, cyld cutaneous syndrome, familial multiple trichoepithelioma, genodermatosis, trichoepithelioma

## Abstract

Familial multiple trichoepithelioma (FMT) is a rare autosomal dominant genodermatosis characterized by the progressive development of multiple benign cutaneous adnexal tumors with follicular differentiation, predominantly involving the central face. FMT is currently recognized as a phenotypic manifestation of CYLD cutaneous syndrome (CCS), a hereditary tumor predisposition disorder associated with pathogenic germline variants in the tumor-suppressor gene CYLD. Cutaneous tumors typically begin to develop around puberty or early adulthood and progressively accumulate over subsequent decades, potentially resulting in substantial facial disfigurement and functional impairment.

We report FMT affecting three consecutive generations of the same family. Case 1, a 74-year-old female, exhibited extensive facial involvement characterized by numerous papules, nodules, and large confluent tumors that had progressively accumulated over several decades, producing marked alteration of facial contours and substantial cosmetic and functional impairment. Case 2, her daughter, presented with multiple centrofacial papules and small nodules, with a considerably lower tumor burden. Case 3, her grandson, also exhibited multiple facial papules, representing the third consecutive clinically affected generation.

Representative facial lesions from all three affected individuals underwent biopsy, with histopathological findings consistent with trichoepithelioma in each case. The specimens demonstrated dermal basaloid proliferations with follicular differentiation and keratin-containing cystic structures. Molecular genetic testing performed exclusively in Case 3 identified the heterozygous pathogenic germline nonsense variant CYLD c.2806C>T, p.(Arg936Ter), previously associated with phenotypes within the CCS spectrum. Cases 1 and 2 did not undergo molecular testing; therefore, segregation of this specific variant across all three generations was not directly established.

Because of the extensive tumor burden in Case 1, staged surgical excision of the largest and most deforming lesions was performed, resulting in substantial postoperative improvement in facial contour and reduction in tumor burden.

This family illustrates the characteristic multigenerational presentation of CCS and the considerable cumulative burden that may develop over decades. Differences in tumor burden across generations should be interpreted cautiously because age-related tumor accumulation represents an important confounding factor. The convergence of clinical morphology, histopathological findings, vertical familial transmission, and molecular confirmation in one affected descendant highlights the importance of clinicopathological correlation, accurate genetic interpretation, individualized treatment, genetic counseling, and long-term dermatologic surveillance.

## Introduction

Trichoepithelioma is a benign cutaneous adnexal neoplasm with follicular differentiation that may occur as a solitary sporadic lesion or, less commonly, as multiple lesions with familial aggregation. The familial presentation, traditionally termed familial multiple trichoepithelioma (FMT), represents a rare genodermatosis predominantly inherited in an autosomal dominant pattern [1-3].

Clinically, FMT is characterized by the progressive development of numerous firm, skin-colored or occasionally erythematous papules and nodules, predominantly distributed over the central face, particularly the nose, nasolabial folds, upper lip, forehead, and periocular regions. Lesions may also involve the scalp and other anatomical sites. They commonly begin to appear during childhood, around puberty, or in early adulthood and tend to increase progressively in number and, in some patients, size throughout life [1,2].

The natural history of the disease is characterized by cumulative tumor development over several decades. Consequently, older affected individuals may exhibit extensive and confluent lesions, whereas younger relatives may present with a comparatively limited number of papules or nodules. This age-dependent accumulation is particularly important when interpreting differences in clinical severity within affected families [1,4].

FMT is currently recognized within the phenotypic spectrum of CYLD cutaneous syndrome (CCS), together with conditions historically designated as Brooke-Spiegler syndrome and familial cylindromatosis. Although these disorders were initially described as separate clinical entities, molecular studies have demonstrated that they represent overlapping manifestations of a hereditary tumor predisposition syndrome associated with pathogenic germline variants in CYLD [1,5,6].

The CCS spectrum includes trichoepitheliomas, cylindromas, and spiradenomas, which may occur individually or in different combinations, even among members of the same family. Accordingly, FMT remains a useful descriptive designation for a predominantly follicular tumor phenotype, whereas CCS more accurately reflects the underlying hereditary disorder [1,6].

The CYLD gene, located on chromosome 16q12.1, encodes a deubiquitinating enzyme with tumor-suppressive functions. CYLD regulates signaling pathways involved in cellular proliferation, differentiation, inflammation, apoptosis, and survival. Loss of normal CYLD function predisposes affected individuals to the development of characteristic cutaneous adnexal neoplasms [1,3,5,7].

Recent molecular studies have further demonstrated that CCS-associated tumors exhibit a distinct molecular landscape involving recurrent alterations in CYLD and additional tumor-associated genes, providing insight into the biological heterogeneity of these neoplasms [8].

Although trichoepitheliomas are histologically benign, their progressive accumulation may cause substantial facial disfigurement, functional impairment, psychological distress, and reduced quality of life. Patients with extensive disease may require repeated therapeutic procedures throughout their lifetime. Complete removal of all lesions may be impractical because of their number, size, distribution, and continued development over time [1,9].

Recognition of a familial pattern is therefore important not only for establishing the diagnosis but also for identifying relatives who may benefit from dermatologic surveillance, genetic counseling, and targeted molecular investigation.

We report three affected members of the same family spanning three consecutive generations, including a 74-year-old proband with extensive, confluent, and disfiguring facial trichoepitheliomas. Representative lesions from all three individuals were histopathologically consistent with trichoepithelioma, while molecular testing identified the previously reported pathogenic germline CYLD variant c.2806C>T, p.(Arg936Ter) in the affected third-generation family member.

This report illustrates the clinical expression of CCS across successive generations, the importance of age-related tumor accumulation when interpreting apparent differences in severity, and the potential benefit of staged surgical excision in patients with advanced tumor burden. It also emphasizes the distinction between clinicopathological evidence of familial disease and direct molecular confirmation in individual family members.

## Case presentation

Case 1: First-generation proband

A 74-year-old female, the first-generation proband, presented with numerous slowly growing facial lesions that had progressively increased in number and size over several decades. The longstanding course was characterized by the gradual development of additional lesions and progressive enlargement of pre-existing tumors. Over time, several lesions became markedly enlarged and confluent, resulting in substantial alteration of facial contours and significant cosmetic and functional impairment.

Dermatologic examination revealed numerous skin-colored to erythematous papules, nodules, and larger tumors, predominantly with smooth surfaces. Several lesions were confluent and considerably larger than the surrounding papules. The lesions were primarily distributed over the central face, involving the forehead, nose, malar regions, nasolabial folds, perioral region, and periocular areas.

The extensive involvement produced marked distortion of the normal facial architecture. Some of the largest tumors exhibited superficial erosions or ulceration. Chronic local trauma, friction, and tumor volume were considered possible contributing factors; however, these mechanisms could not be established definitively from the available documentation. Importantly, superficial ulceration was not considered sufficient to establish or exclude malignant transformation (Figure 1).

**Figure 1:**
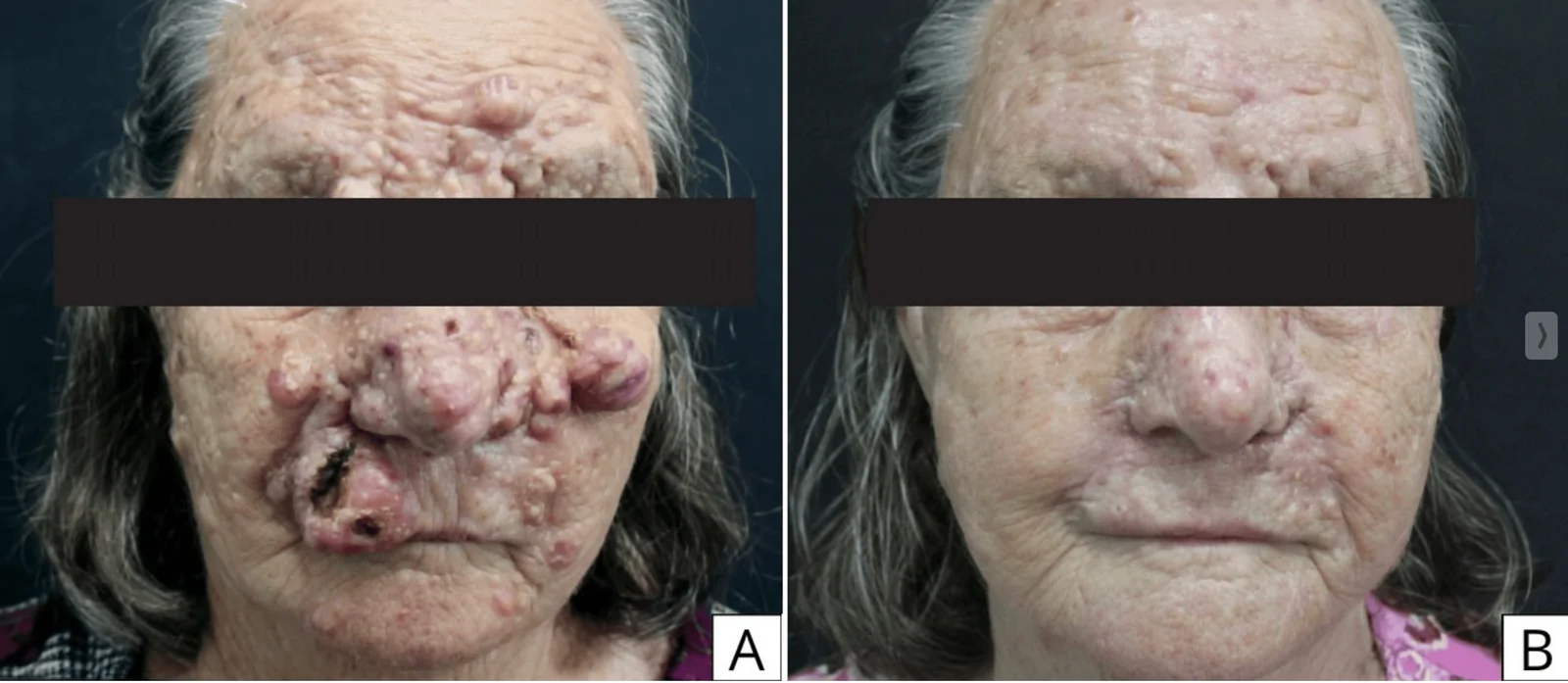
Clinical presentation and outcome after surgical treatment. (A) Extensive facial involvement with numerous papules, nodules, and large confluent tumors causing marked distortion of the facial contours. (B) Clinical appearance after staged surgical excision of the largest lesions, demonstrating substantial reduction in tumor burden and improvement in facial contour.

A representative facial lesion underwent biopsy. Histopathological examination demonstrated a dermal proliferation composed of basaloid epithelial aggregates with follicular differentiation and multiple keratin-containing cystic structures. Low-power examination demonstrated the overall architecture of the dermal proliferation, while higher-power examination revealed basaloid cell aggregates associated with keratinizing cystic structures. These findings supported the diagnosis of trichoepithelioma (Figure 2).

**Figure 2:**
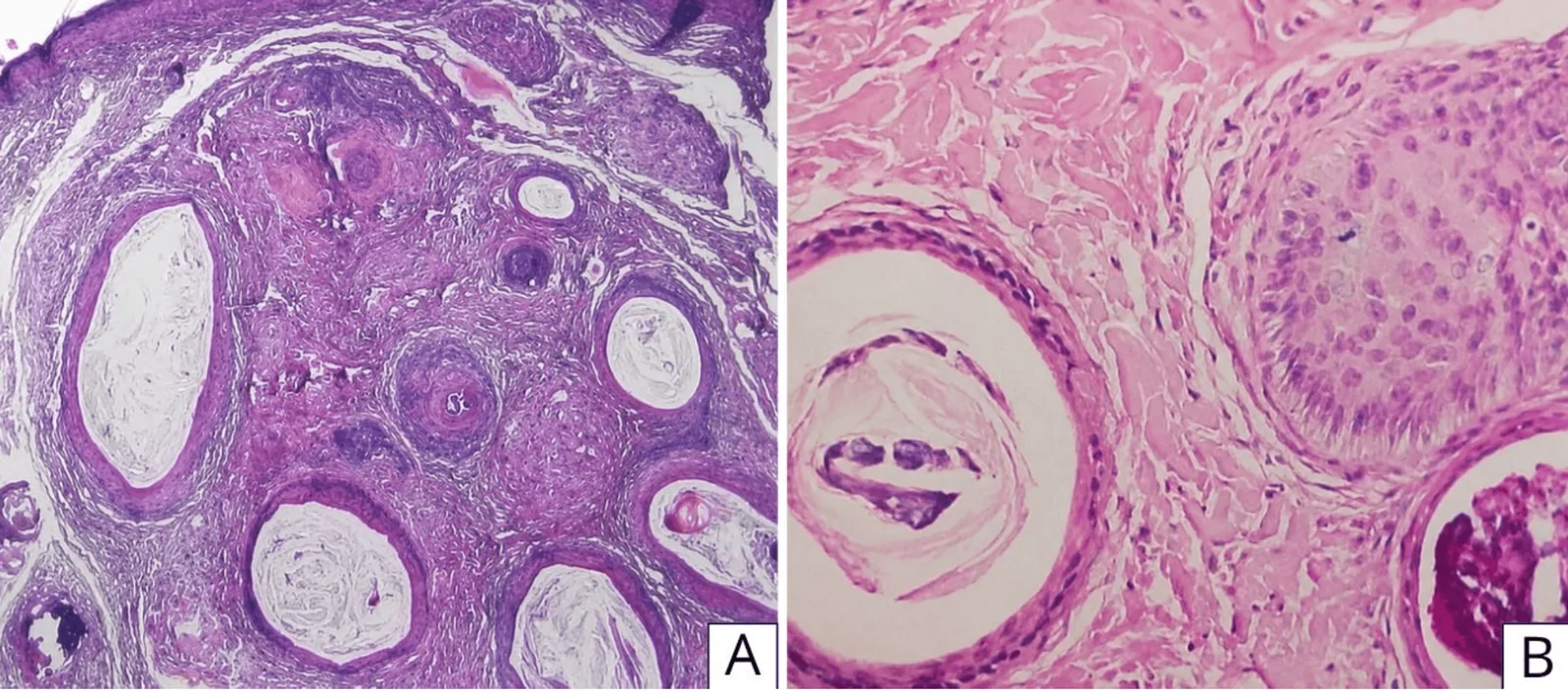
Histopathological examination of a facial lesion from the first-generation proband (grandmother). (A) Low-power view demonstrating a dermal basaloid proliferation with follicular differentiation and multiple keratin-containing cystic structures (hematoxylin and eosin). (B) Higher-power view demonstrating basaloid cell aggregates associated with keratinizing cystic structures, supporting follicular differentiation and the diagnosis of trichoepithelioma.

The combination of characteristic follicular differentiation, keratin-containing cystic structures, extensive centrofacial involvement, and familial aggregation supported the diagnosis of multiple trichoepitheliomas within the CCS spectrum.

The available histopathological documentation establishes the diagnosis in the representative sampled lesion. However, the precise anatomical biopsy site and whether the ulcerated lesions underwent separate histopathological examination could not be reliably established from the available information. Therefore, the histological findings should not be interpreted as confirming that every facial tumor was a trichoepithelioma or that malignant transformation was excluded in all lesions.

Given the extensive tumor burden and resulting functional and cosmetic impairment, a staged surgical approach was selected. Complete removal of all lesions in a single procedure was considered impractical because of their number, size, and anatomical distribution.

The largest and most deforming lesions were prioritized for excision while preserving as much uninvolved tissue as possible. The surgical strategy was directed toward progressive reduction of tumor burden and improvement of facial contour and function rather than complete removal of every lesion.

The documented postoperative evolution was satisfactory, with substantial reduction in tumor burden and visible improvement in facial contour (Figure 1B).

The available information does not permit reliable reconstruction of the exact number of surgical stages, all operative details, or a standardized long-term follow-up interval. Accordingly, the observed postoperative improvement is reported without implying complete disease clearance, absence of recurrence, or established long-term surgical efficacy.

Case 1 did not undergo molecular genetic testing. Therefore, although the clinical phenotype, histopathological findings, and family history strongly support CCS, the presence of the specific CYLD variant identified in Case 3 was not directly demonstrated in this individual.

Case 2: Second-generation affected family member

The daughter of Case 1 represented the second consecutive affected generation. She presented with multiple facial papules and small nodules predominantly involving the central face.

Her phenotype was clinically similar to that of Case 1, with a characteristic centrofacial distribution of lesions. However, the tumor burden was substantially less extensive at the time of evaluation, and the lesions were predominantly smaller, without the large confluent tumor masses observed in her mother (Figure 3).

**Figure 3:**
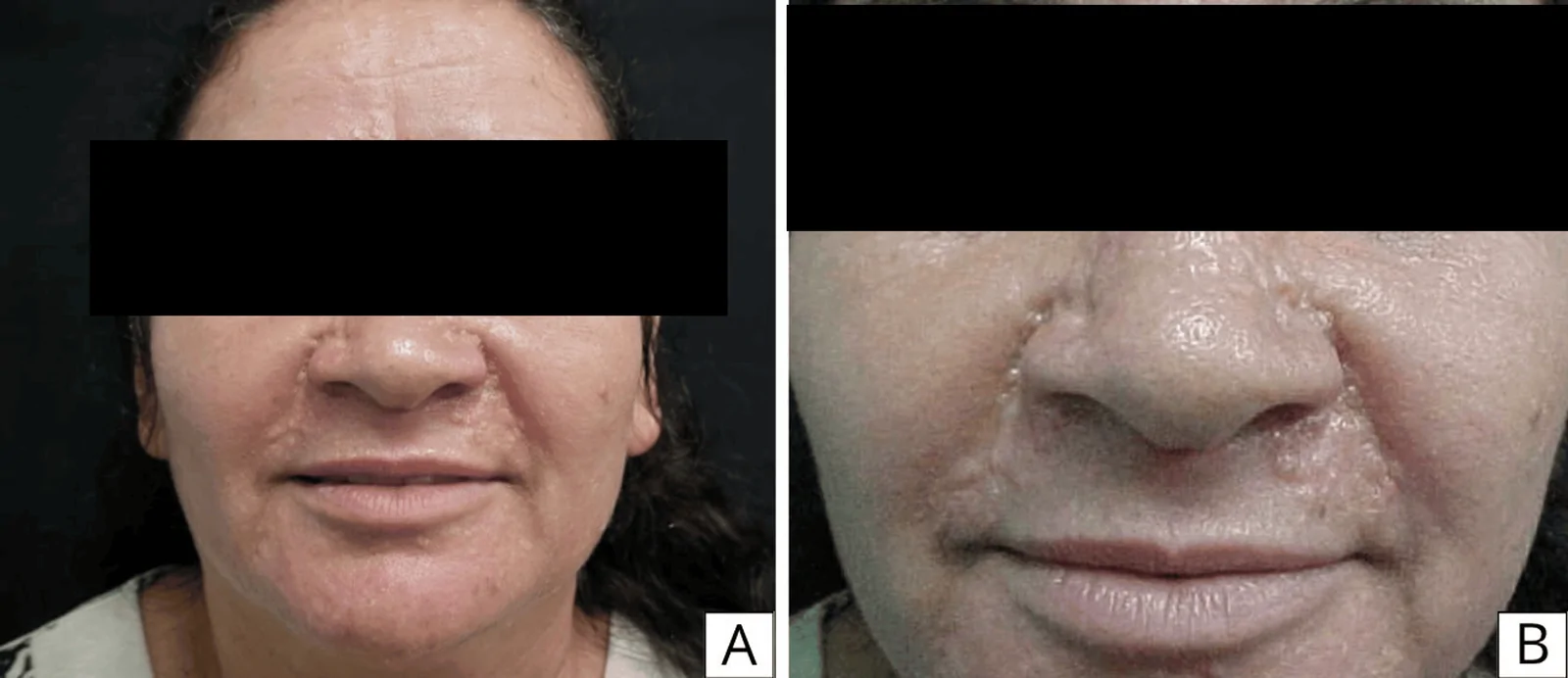
Phenotypic expression of familial multiple trichoepithelioma in the second generation. (A) Frontal view of the affected second-generation family member showing multiple facial papules and nodules. (B) Closer view highlighting the centrofacial distribution of the lesions, consistent with familial multiple trichoepithelioma.

A representative facial lesion underwent biopsy. Histopathological examination demonstrated a dermal basaloid epithelial proliferation with follicular differentiation and keratin-containing cystic structures, consistent with trichoepithelioma (Figure 4).

**Figure 4:**
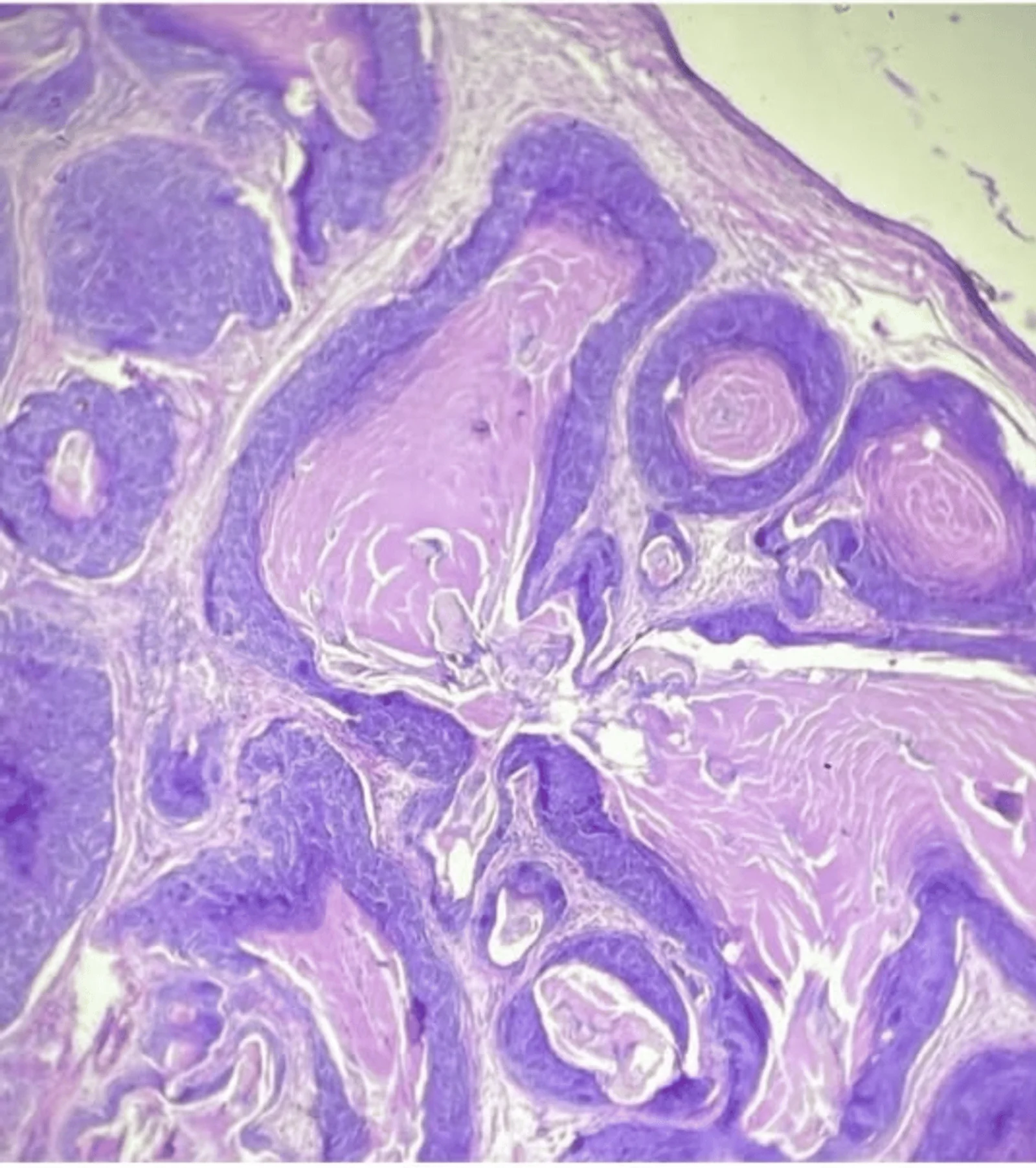
Histopathological findings in Case 2. Representative facial lesion from Case 2 showing a dermal basaloid epithelial proliferation with follicular differentiation and keratin-containing cystic structures, consistent with trichoepithelioma (hematoxylin and eosin staining).

The precise age at lesion onset, current age at evaluation, disease duration, associated symptoms, previous treatments, and standardized longitudinal follow-up were not available in the documentation used for this report. Consequently, the clinical comparison with Case 1 is descriptive and should not be interpreted as a quantitative assessment of disease progression or intrinsic severity.

The lower tumor burden observed in Case 2 may reflect differences in age, cumulative disease duration, or intrinsic phenotypic expression. The relative contribution of these factors cannot be determined from the available cross-sectional information.

Case 2 did not undergo molecular genetic testing. Therefore, although the clinical phenotype, histopathological findings, and familial pattern strongly support CCS, the presence of the specific CYLD variant identified in Case 3 remains unconfirmed in this individual.

Case 3: Third-generation affected family member

The grandson of Case 1 represented the third consecutive affected generation. The characteristic distribution and morphology of the lesions were concordant with the phenotype observed in his mother and grandmother. Dermatologic examination demonstrated multiple facial papules predominantly involving the central face, with a lower tumor burden at the time of evaluation compared with the older affected family members (Figure 5).

**Figure 5:**
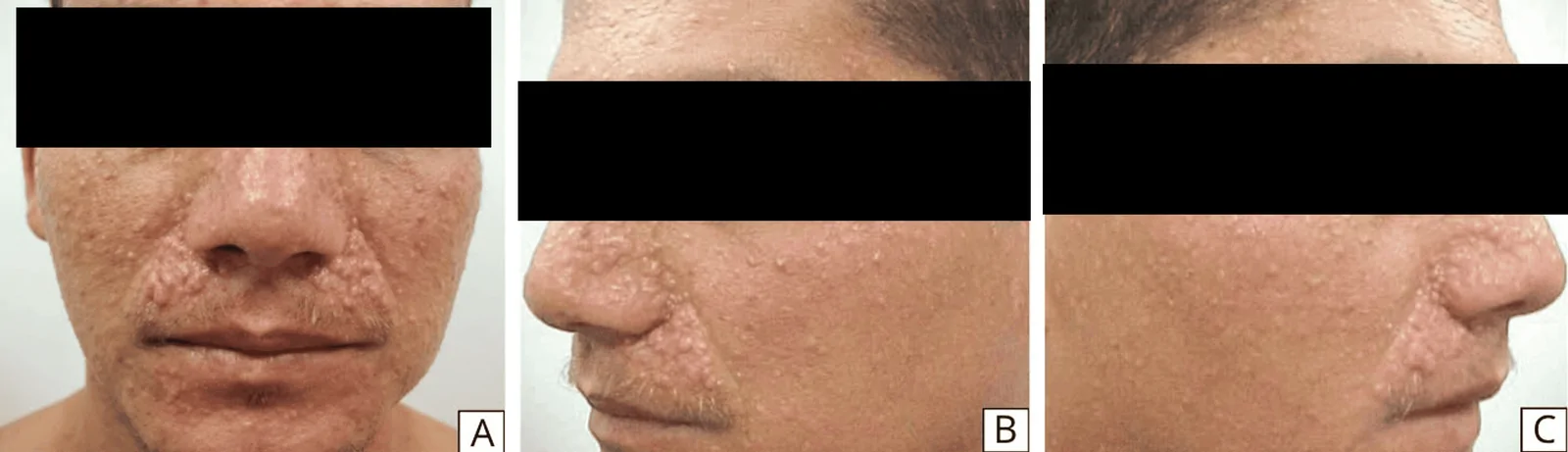
Phenotypic expression of familial multiple trichoepithelioma in Case 3 (third generation). Affected third-generation family member, the grandson of Case 1, showing multiple facial papules with a less extensive tumor burden at the time of evaluation.

A representative facial lesion underwent biopsy. Histopathological examination demonstrated a dermal basaloid epithelial proliferation with follicular differentiation and keratin-containing cystic structures, consistent with trichoepithelioma (Figure 6).

**Figure 6:**
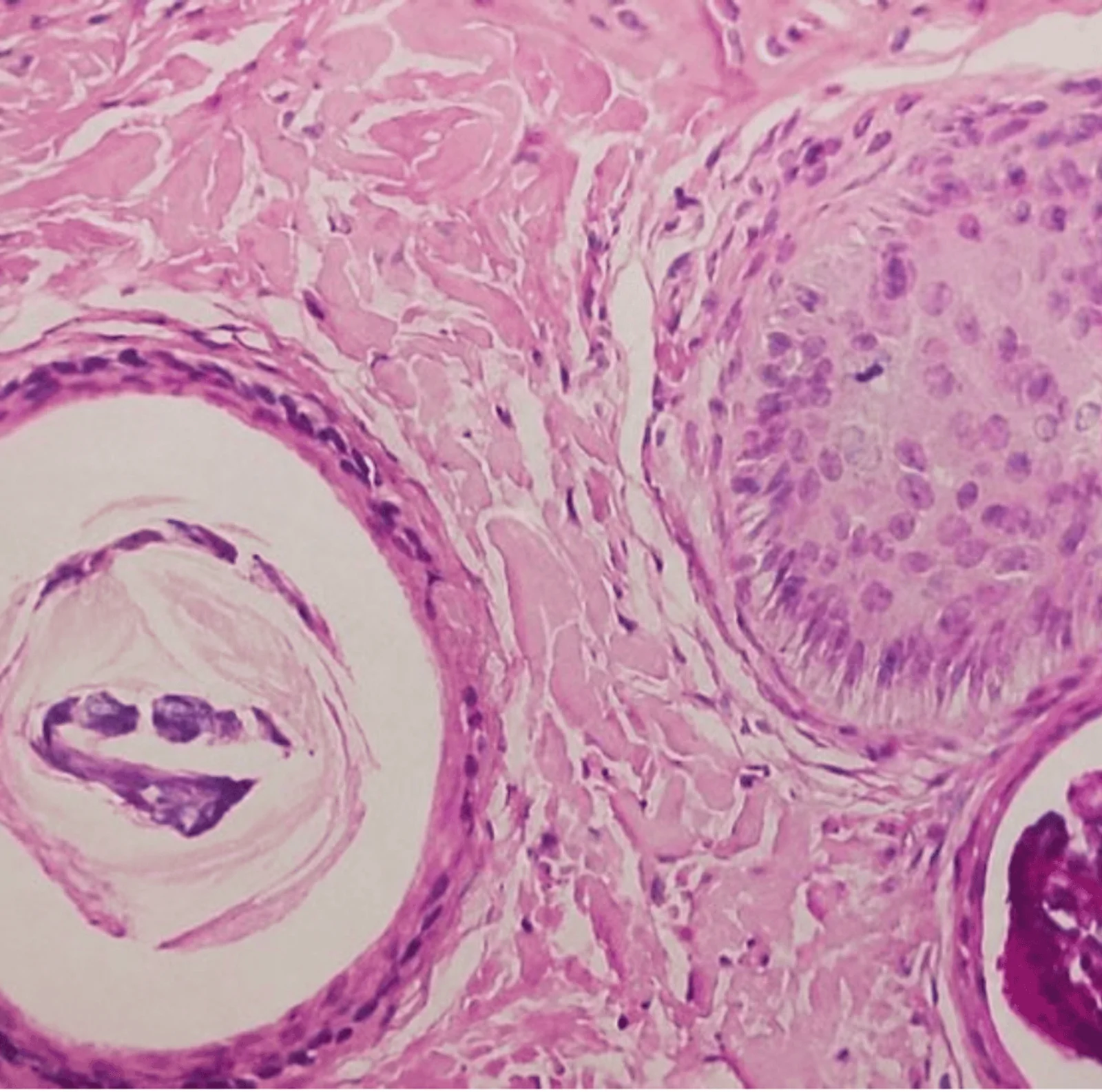
Histopathological findings in Case 3. Histopathological examination of a representative facial lesion demonstrating dermal aggregates of basaloid epithelial cells with follicular differentiation and keratin-containing cystic structures, consistent with trichoepithelioma (hematoxylin and eosin staining).

As in Case 2, precise chronological information regarding the age at lesion onset, disease duration, symptoms, previous treatments, and longitudinal follow-up was not available in the documentation used for this report. Therefore, differences in tumor burden across generations are presented as descriptive clinical observations rather than evidence of progressively milder disease.

Because of the characteristic clinical presentation and the occurrence of similar lesions in three consecutive generations, molecular genetic testing was performed in Case 3. The analysis identified the heterozygous pathogenic germline nonsense variant CYLD c.2806C>T, p.(Arg936Ter).

The CYLD c.2806C>T, p.(Arg936Ter) variant introduces a premature termination codon near the C-terminus of the protein and is predicted to disrupt the final 21 amino acids. This variant has previously been reported in association with familial cylindromatosis, Brooke-Spiegler syndrome, and multiple familial trichoepithelioma. It is also documented in the ClinVar database (Variation ID: 5259; accession VCV000005259), with pathogenic/likely pathogenic classifications reported by multiple laboratories without conflicting classifications. Therefore, its identification in the present case represents molecular confirmation of a previously described disease-associated alteration rather than the discovery of a novel variant. The present report does not claim an original submission to ClinVar or another public variant database.

Although the available molecular information supports the pathogenic classification, the detailed sequencing methodology and specific American College of Medical Genetics and Genomics and the Association for Molecular Pathology (ACMG/AMP) evidence criteria applied by the laboratory responsible for Case 3 were not available for independent review. Accordingly, no undocumented laboratory methodology or classification criteria are inferred [10].

Identification of the heterozygous pathogenic germline CYLD variant establishes molecular confirmation of CCS specifically in Case 3.

Although the highly concordant clinical and histopathological findings in Cases 1 and 2 strongly support the same hereditary disorder, molecular segregation of the specific variant across the three generations was not directly demonstrated.

Familial correlation

The occurrence of characteristic centrofacial lesions in a grandmother, her daughter, and her grandson demonstrated vertical familial involvement across three consecutive generations, strongly consistent with an autosomal dominant inheritance pattern.

Representative lesions from all three affected family members were histopathologically consistent with trichoepithelioma. The molecular identification of a pathogenic germline CYLD variant in Case 3 provided additional evidence for the hereditary basis of the disease. However, because Cases 1 and 2 did not undergo genetic testing, carriage of the same specific variant in these individuals cannot be considered molecularly established. The concordant clinical phenotypes, histopathological findings, and vertical familial transmission nevertheless strongly support FMT within the CCS spectrum.

The three-generation familial relationship is represented schematically in Figure 7.

**Figure 7:**
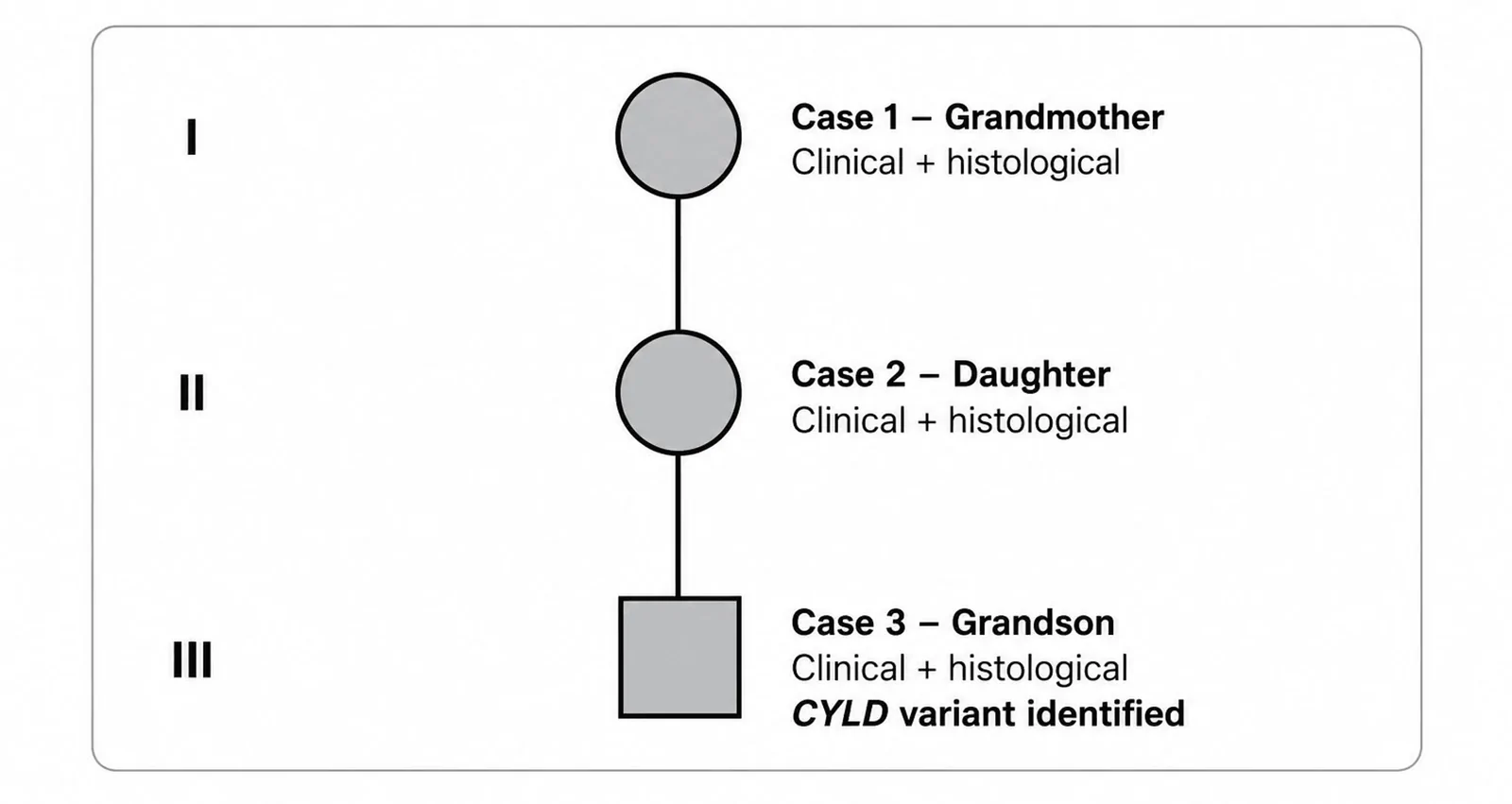
Three-generation lineage schematic of familial multiple trichoepithelioma. Simplified pedigree illustrating three consecutive generations affected by familial multiple trichoepithelioma within the CYLD cutaneous syndrome spectrum. Case 1 (grandmother), Case 2 (daughter), and Case 3 (grandson) presented with characteristic clinical manifestations and histopathological findings consistent with trichoepithelioma. Molecular testing identified the heterozygous pathogenic germline CYLD variant c.2806C>T, p.(Arg936Ter) exclusively in Case 3. Cases 1 and 2 were not genetically tested. Filled symbols indicate clinically affected individuals; circles represent females, and the square represents a male. The schematic includes only the three reported individuals and does not establish the clinical or genetic status of other relatives.

The identification of a pathogenic familial variant has important implications for affected and at-risk relatives. Targeted genetic testing of Cases 1 and 2 would be appropriate to investigate segregation of the variant, while other potentially at-risk relatives may benefit from genetic counseling and individualized dermatologic surveillance.

Because actual counseling outcomes and additional molecular testing were not documented in the available information, these measures are presented as clinical recommendations rather than procedures confirmed to have been performed.

Long-term dermatologic surveillance is recommended because additional lesions may develop over time and changes in the morphology or behavior of individual tumors may warrant clinical reassessment and histopathological examination.

## Discussion

The present report illustrates several clinically relevant features of CCS through the evaluation of three affected members of the same family. Its principal findings include extensive cumulative facial tumor burden in the oldest affected individual, a concordant adnexal tumor phenotype across three consecutive generations, histopathological findings consistent with trichoepithelioma in representative lesions from all three family members, identification of a previously reported pathogenic germline CYLD variant in one affected descendant, and substantial postoperative improvement following staged surgical excision in the most severely affected patient.

Historically, FMT, Brooke-Spiegler syndrome, and familial cylindromatosis were regarded as distinct disorders based on their predominant clinical and histopathological manifestations. Increasing molecular evidence has demonstrated that these phenotypes are associated with pathogenic germline variants in the same tumor-suppressor gene and may coexist within individual families [1,2]. They are therefore now understood as overlapping phenotypic expressions of CCS [3,5].

The CCS spectrum encompasses trichoepitheliomas, cylindromas, and spiradenomas, which may occur in different combinations and with variable severity. The predominant tumor type may differ among affected relatives. Consequently, the term FMT remains clinically useful for describing patients with predominantly multiple trichoepitheliomas, while CCS represents the broader molecularly defined hereditary syndrome [7,8].

CCS follows an autosomal dominant inheritance pattern. An individual heterozygous for a pathogenic germline CYLD variant has a 50% probability of transmitting the variant to each offspring. Penetrance is generally considered high but may be age-dependent, and clinical expression can vary substantially among affected individuals, including members of the same family [6,9,11].

The natural history of CCS is particularly relevant when interpreting disease burden across generations. Cutaneous tumors commonly begin to appear around puberty or early adulthood and progressively accumulate over subsequent decades. Although the rate of tumor development varies, older individuals may exhibit substantially greater numbers of lesions and larger tumors simply because they have experienced a longer period of tumor formation [4,12].

This cumulative pattern is illustrated by the present family. Case 1, aged 74 years, exhibited extensive facial involvement with large, confluent, and disfiguring tumors, whereas Cases 2 and 3 demonstrated substantially lower tumor burdens at the time of evaluation.

These differences should not be interpreted as evidence of progressively milder disease across generations. Age-related tumor accumulation, differences in disease duration, and potential intrafamilial phenotypic variability are important confounding factors. Because precise onset ages and standardized longitudinal measurements were unavailable, the observed differences in tumor burden are best interpreted as descriptive cross-sectional findings rather than quantitative evidence of differences in intrinsic disease severity [13].

Nevertheless, the coexistence of characteristic centrofacial lesions across three consecutive generations, together with histopathological confirmation in all affected individuals and molecular identification of a pathogenic CYLD variant in Case 3, provides strong evidence of familial CCS.

This distinction is clinically important because younger affected relatives with a relatively limited phenotype should not necessarily be reassured that their condition will remain mild. Additional tumors may continue to emerge, and pre-existing lesions may enlarge over time. Longitudinal dermatologic surveillance is therefore appropriate [14].

At the molecular level, CYLD encodes a tumor-suppressive deubiquitinating enzyme involved in the regulation of signaling pathways controlling cellular proliferation, differentiation, survival, apoptosis, and inflammatory responses [13,14]. Loss of CYLD function predisposes affected individuals to the development of characteristic cutaneous adnexal tumors. The biological consequences of CYLD dysfunction are complex and extend beyond a single signaling pathway. Integrative molecular profiling of CCS-associated tumors has demonstrated a relatively low overall mutational burden, recurrent alterations involving CYLD, DNMT3A, and BCOR, and molecular differences among the adnexal tumor types occurring within the syndrome [8]. These findings reinforce the concept that germline CYLD dysfunction establishes an inherited susceptibility background upon which additional molecular events contribute to the development and biological heterogeneity of individual tumors.

The molecular finding in Case 3 represents an important component of this report. The identified heterozygous germline CYLD c.2806C>T, p.(Arg936Ter) variant introduces a premature termination codon near the C-terminus of the protein and is predicted to eliminate the final 21 amino acids. The same variant has previously been reported in association with different phenotypes within the CCS spectrum, including familial cylindromatosis, Brooke-Spiegler syndrome, and multiple familial trichoepithelioma [13]. Its identification in the present family therefore does not represent a novel molecular discovery. Instead, it provides molecular confirmation of an established disease-associated variant in an affected individual with a characteristic clinical and histopathological phenotype.

The occurrence of different clinical manifestations associated with the same pathogenic CYLD alteration further supports the absence of a straightforward genotype-phenotype correlation. The molecular variant alone cannot reliably predict the number, distribution, histological type, or eventual severity of tumors in a particular carrier [11,12,14]. Frameshift and nonsense variants resulting in premature protein truncation constitute a substantial proportion of reported pathogenic germline alterations in CCS. More than 100 family-specific pathogenic variants have been described, with relatively few recurrent mutational hotspots [1,14].

Consequently, molecular findings should be interpreted in conjunction with the clinical phenotype, histopathological findings, and family history rather than used independently to predict disease severity.

The molecular result also requires precise attribution. In this family, Case 3 was the only individual who underwent genetic testing. Therefore, molecular confirmation of the pathogenic CYLD variant applies specifically to Case 3.

The concordant clinical and histopathological findings in Cases 1 and 2, together with the vertical pattern of familial involvement, strongly support a shared hereditary disorder. However, the presence of the identical variant in these two individuals remains unverified.

Targeted testing for the identified familial variant would provide the most direct means of establishing molecular segregation across the three generations. This distinction is important when reporting hereditary disorders because clinical concordance and pedigree structure, although highly informative, should not be presented as equivalent to direct molecular confirmation.

The lack of detailed documentation regarding the sequencing methodology and the specific ACMG/AMP criteria applied by the original testing laboratory is an additional methodological limitation. Although the identified variant has previously been reported as pathogenic and associated with CCS, no undocumented laboratory classification criteria should be retrospectively assigned [10].

From a histopathological perspective, trichoepitheliomas demonstrate follicular differentiation and typically contain dermal aggregates or nests of basaloid epithelial cells, horn or keratin cysts, and structures resembling primitive follicular components. A specialized fibrocytic stroma and follicular papillary mesenchymal bodies may also be present [2,3].

The principal histopathological differential diagnosis is basal cell carcinoma (BCC), particularly in isolated lesions, small biopsy specimens, or tumors in which follicular differentiation is subtle. Both trichoepithelioma and BCC may contain basaloid epithelial proliferations. However, trichoepithelioma more typically exhibits conspicuous follicular differentiation, keratin-containing cysts, follicular papillary mesenchymal structures, and a relatively organized relationship between epithelial aggregates and the surrounding fibrous stroma. In contrast, BCC may demonstrate peripheral nuclear palisading, stromal retraction clefts, mucinous stromal changes, and infiltrative or destructive growth patterns, depending on the histological subtype.

These findings are not individually absolute, and morphological overlap may occur. Consequently, accurate differentiation requires evaluation of the overall architectural and cytological findings together with clinical context.

In the present cases, the documented findings of basaloid epithelial proliferations associated with follicular differentiation and keratin-containing cystic structures supported the diagnosis of trichoepithelioma.

The characteristic multiplicity of lesions, predominantly centrofacial distribution, and involvement of three consecutive generations further strengthened the clinicopathological interpretation.

Ancillary immunohistochemical markers, including CK20, CD10, and BCL2, may be useful in selected diagnostically challenging cases, particularly when conventional histopathological findings are equivocal. However, their interpretation depends on staining distribution and morphological context, and immunohistochemistry is not invariably necessary when routine histopathology and clinicopathological correlation are sufficiently characteristic.

The available documentation does not establish that ancillary immunohistochemical studies or independent second dermatopathological review were performed in the present cases. These investigations are therefore not claimed.

Importantly, histopathological confirmation of representative lesions does not establish the identity of every tumor in an individual with numerous facial lesions. Separate assessment is warranted when a lesion demonstrates atypical morphology or concerning clinical behavior. This consideration is particularly relevant to Case 1, in whom some large tumors exhibited superficial erosion or ulceration.

Although chronic trauma, friction, and tumor volume may contribute to ulceration in large benign lesions, these mechanisms cannot be assumed without appropriate clinical assessment. Ulceration may also occur in malignant tumors, and suspicious changes should prompt consideration of histopathological examination.

Because the available documentation does not establish whether the ulcerated tumors were specifically sampled or whether every excised lesion underwent histopathological assessment, malignant transformation cannot be considered definitively excluded in all lesions.

Most CYLD-associated cutaneous tumors are benign; however, malignant transformation has been reported within the CCS spectrum [1,6]. Accordingly, long-term dermatologic surveillance should include careful assessment for rapid or disproportionate growth, persistent spontaneous ulceration, bleeding, new pain, fixation, or significant changes in the morphology of previously stable lesions. Such findings should prompt clinical reassessment and histopathological examination when indicated.

Management of extensive FMT remains challenging. Complete removal of every lesion may be impractical in patients with a high tumor burden, particularly because additional tumors can continue to develop throughout life. Treatment should therefore be individualized according to lesion size, anatomical location, symptoms, functional consequences, cosmetic impact, diagnostic uncertainty, suspicion of malignant transformation, and patient preference [1,9]. Surgical excision remains an important therapeutic option for larger, symptomatic, diagnostically uncertain, or disfiguring lesions. In extensively affected patients, staged procedures can progressively reduce tumor burden while limiting the extent of each operation and preserving uninvolved tissue. Other approaches, including electrosurgery and ablative laser techniques, have been used for selected smaller lesions, although incomplete removal, recurrence, scarring, and continued development of new tumors remain important limitations [1,9]. A staged surgical approach has previously been described as a practical management strategy for extensive familial trichoepithelioma [9].

The present case further illustrates the potential value of this approach in patients with substantial facial involvement.

In Case 1, the large number, size, and distribution of tumors made complete removal during a single procedure impractical. Prioritization of the largest and most deforming lesions allowed progressive reduction of tumor burden while preserving uninvolved facial tissue.

The documented postoperative appearance demonstrated substantial improvement in facial contour, supporting the potential benefit of individualized sequential surgical management in advanced disease. Nevertheless, the available information does not establish a standardized long-term follow-up interval or permit conclusions regarding recurrence rates or durable tumor control. Accordingly, the surgical outcome should be interpreted as documented postoperative improvement rather than evidence of definitive or curative treatment.

Molecular diagnosis also has implications extending beyond the tested individual. Once a pathogenic familial variant has been identified, genetic counseling can address the autosomal dominant inheritance pattern, the 50% probability of transmission to offspring, variable expressivity, age-dependent clinical manifestations, and the availability of targeted testing for at-risk relatives [1,14].

In the present family, Cases 1 and 2 would be appropriate candidates for targeted testing of the CYLD c.2806C>T, p.(Arg936Ter) variant identified in Case 3, provided that testing is available and consistent with their preferences. Other potentially at-risk relatives may also benefit from pedigree-based risk assessment, dermatologic examination, and genetic counseling. Testing decisions should consider age, clinical manifestations, family relationships, and the potential implications of identifying a pathogenic hereditary variant. For clinically affected individuals, molecular confirmation may clarify the hereditary diagnosis and facilitate cascade testing. For currently unaffected relatives, counseling should explain that the absence of visible lesions at a particular age does not necessarily exclude variant carriage because clinical manifestations may develop later.

The available documentation does not permit confirmation of specific counseling outcomes or additional targeted genetic testing among relatives. Accordingly, these measures are presented as evidence-based recommendations rather than interventions established to have occurred. Longitudinal dermatologic surveillance remains appropriate because new tumors may develop throughout life and individual lesions may require treatment or histopathological reassessment.

An important strength of this report is the documentation of a clinically concordant adnexal tumor disorder across three consecutive generations. The extensive phenotype of the oldest affected family member provides a striking illustration of the potential cumulative burden of FMT over several decades, whereas the less extensive manifestations in the younger generations emphasize the difficulty of separating age-related tumor accumulation from intrinsic phenotypic variability. Additional strengths include histopathological confirmation of trichoepithelioma in representative lesions from all three affected individuals, identification of a previously established pathogenic CYLD variant in one affected descendant, and documentation of improvement following staged surgical treatment in the most severely affected patient.

This report also has limitations. Molecular testing was performed exclusively in Case 3, preventing direct demonstration of segregation of the pathogenic variant across all three generations.

Histopathological examination was based on representative sampled lesions and therefore cannot establish the histological identity of every facial tumor.

The available information does not permit reliable reconstruction of precise lesion onset ages, standardized lesion counts, detailed longitudinal disease measurements, or complete treatment histories for the younger affected family members. Similarly, the exact histopathological assessment of ulcerated tumors, all operative details, and standardized long-term postoperative follow-up could not be established from the available documentation. Detailed molecular testing methodology and the individual ACMG/AMP evidence criteria applied by the original laboratory were also unavailable for independent review.

These limitations restrict quantitative comparisons of disease progression, conclusions regarding the histological nature of unsampled tumors, and assessment of long-term therapeutic outcomes. However, they do not negate the principal documented observations: characteristic centrofacial lesions across three consecutive generations, histopathological findings consistent with trichoepithelioma in all three affected individuals, molecular identification of a pathogenic germline CYLD variant in Case 3, and visible postoperative improvement following staged surgical excision in Case 1.

Despite these limitations, the convergence of clinical morphology, characteristic distribution, vertical familial transmission, representative histopathological findings, and molecular confirmation in an affected descendant strongly supports FMT within the CCS spectrum in this family. The present report emphasizes the importance of recognizing hereditary patterns in patients with multiple adnexal tumors. Such recognition may facilitate appropriate molecular investigation, genetic counseling, individualized treatment, and long-term surveillance of affected and at-risk relatives.

## Conclusions

FMT is a rare autosomal dominant genodermatosis within the phenotypic spectrum of CCS and should be considered when multiple centrofacial papules and nodules occur in individuals with similarly affected relatives. The present report describes three affected members of the same family spanning three consecutive generations. The 74-year-old first-generation proband exhibited extensive, confluent, and disfiguring facial tumors, whereas the second- and third-generation family members demonstrated less extensive centrofacial disease at the time of evaluation. Representative lesions from all three affected individuals were histopathologically consistent with trichoepithelioma. Molecular testing identified the previously reported pathogenic germline CYLD c.2806C>T, p.(Arg936Ter) variant exclusively in Case 3. The specific variant was not directly investigated in Cases 1 and 2, and molecular segregation across the family therefore remains unconfirmed. Differences in tumor burden across generations illustrate the variable clinical presentation of CCS but should not be interpreted as evidence of progressively milder disease. Age-related tumor accumulation, differences in disease duration, and the absence of standardized longitudinal measurements limit direct comparisons of severity.

The clinical and histopathological findings across three generations, combined with molecular confirmation in one affected descendant, provide strong evidence of familial CCS. Recognition of the familial pattern has important implications beyond the individual patient. Clinicopathological correlation, molecular investigation when available, genetic counseling, targeted testing of at-risk relatives, and long-term dermatologic surveillance are essential considerations in management. In patients with extensive tumor burden, staged surgical excision may provide meaningful functional and cosmetic improvement while preserving uninvolved tissue and allowing further treatment when necessary. These cases emphasize the value of careful dermatologic examination not only for diagnosing individual tumors but also for recognizing hereditary tumor predisposition syndromes and identifying relatives who may benefit from earlier evaluation, counseling, and longitudinal surveillance.
